# Atrophying Pityriasis Versicolor: A Rare Presentation of a Common Dermatological Disorder and Literature Review

**DOI:** 10.1155/crdm/4558804

**Published:** 2026-04-23

**Authors:** Abdulrahman M. Almalki, Sultan A. Jaafari, Hasan Y. Hannani, Khalid M. Alattas, Radwan A. Abutaleb, Amr M. gamaleldin, Abdulrahman K. Daak

**Affiliations:** ^1^ Department of Dermatology, King Fahad Central Hospital, Jazan, Saudi Arabia; ^2^ Department of Dermatology, Jazan General Hospital, Jazan, Saudi Arabia

**Keywords:** atrophy, atrophying pityriasis versicolor, fungal infections, hypopigmentation, *Malassezia*, pityrosporum, tinea versicolor, yeast

## Abstract

Pityriasis versicolor is a superficial fungal infection of the skin caused by fungi of the genus *Malassezia*. Typically, patients present with well‐defined, hypopigmented, scaly macules, or patches over seborrheic areas. However, rare presentations include papular, confetti‐like spots, and folliculocentric, atrophic, and inverse forms. Diagnosis may be established clinically with the help of noninvasive diagnostic tools such as dermoscopy, Wood’s lamp, and direct microscopic examinations. However, skin biopsies are imperative in equivocal cases. To date, 34 cases have been reported. This study presents a rare presentation of atrophying skin lesions in a patient with pityriasis versicolor.

## 1. Introduction

Also known as tinea versicolor, pityriasis versicolor (PV) is a superficial fungal infection of the skin caused by species of *Malassezia* (formerly known as *Pityrosporum*) [1]. *Malassezia* fungi are dimorphic lipophilic commensal yeasts that are normally found in the flora of normal healthy skin in minute amounts. However, they change to a pathogenic hyphal or mycelial form in certain conditions, such as oily skin, sweating, hot or humid weather, chronic use of corticosteroids, immunodeficiency, or genetic predisposition [2].

PV occurs worldwide, but the prevalence is as high as 50% in tropical countries due to warm and humid weather [1]. The typical clinical presentation is asymptomatic but may include poorly to well‐demarcated hypo‐ or hyperpigmented macules or patches, as well as fine scaly skin, which are distributed over mostly the trunk, neck, shoulders, and upper arms [1–3]. However, other rare clinical presentations have been reported, such as papular, confetti‐like spots; folliculocentric, atrophic, inverse forms; annular hyperpigmented, hyperkeratotic forms extending to the lower limbs; facial localization; and infant forms [2, 4].

In this paper, we present an atypical clinical presentation of PV that manifested with atrophying hyperpigmented macules and patches. This condition can be easily misdiagnosed if not considered among the differential diagnoses of atrophic skin lesions. In addition, we present a comprehensive literature review of the reported cases to increase recognition by clinicians and improve the understanding of the possible hypotheses behind the atrophic form.

## 2. Case Report

**FIGURE 1:**
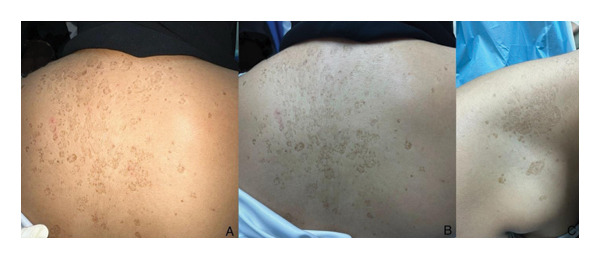
Initial clinical presentation of the skin lesions over the back: (A) with camera flash and (C) without. Similar skin lesions were seen over the upper chest and right side of the neck (C).

A 15‐year‐old female presented to the clinic with complaints of skin lesions on the upper back, shoulders, and upper chest, which had persisted for 2 years and were increasing in size. The patient had not sought any medical care for these skin lesions before and did not use any over‐the‐counter treatments. The patient had no history of joint or muscle weakness and oral or genital lesions. She had no family history of such lesions. Examination revealed numerous well‐defined, oval‐to‐round brown macules of varying sizes, some scattered and others confluent, on the back and shoulders. In addition, multiple plaques of varying sizes and shapes were present on the back, shoulders, and upper chest. All lesions were depressed with abrupt borders and exhibited fine central scaling, giving the appearance of atrophic skin lesions. The surrounding skin was normal. On palpation, the lesions were nonherniated and lacked induration. Zireli’s sign was positive on gentle scraping. Examination of the hair, nails, and mucous membranes was unremarkable. Review of systems was noncontributory (Figure 1). The possible diagnoses included atrophying PV, primary anetoderma, atrophoderma of Pasini and Pierini, morphea, parapsoriasis, mycosis fungoides, dermatomyositis, and lupus erythematosus.

**FIGURE 2:**
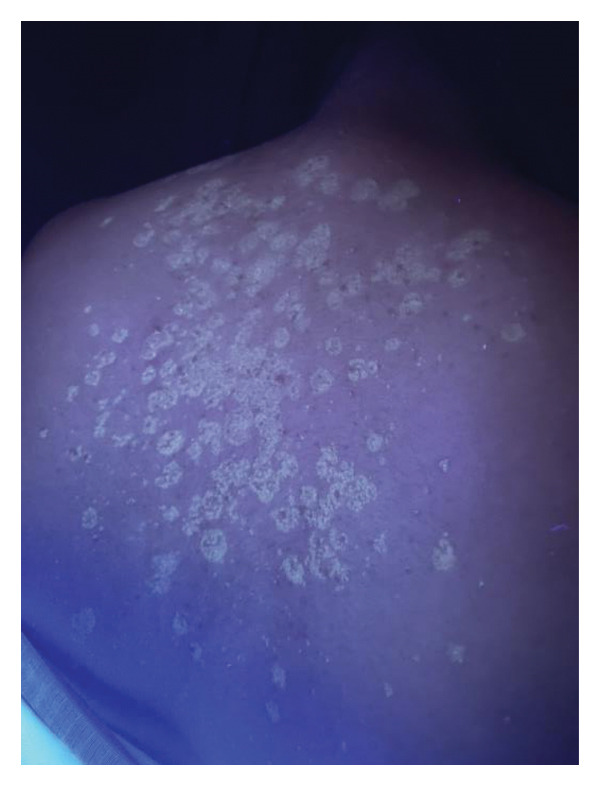
Wood’s lamp examination.

**FIGURE 3:**
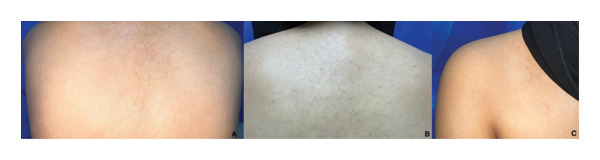
Skin lesions and atrophy completely resolved after 3 months of follow‐up.

Wood’s lamp examination revealed yellow‐green fluorescence (Figure 2). A scraping of the skin lesions was obtained for microscopic examination using a potassium hydroxide (KOH) preparation, which showed multiple short hyphae and spores. Based on these findings, the patient was diagnosed with atrophying PV, and no further investigation was needed. The patient was treated topically with 2% ketoconazole shampoo 3 times/week and 1% terbinafine cream twice daily (both for 6 months), as well as oral itraconazole capsules at 200 mg once daily for 1 month. By 3 months of follow‐up, the lesions had disappeared, and the atrophy had completely resolved (Figure 3). No recurrence was observed 6 months later.

## 3. Discussion

Atrophying PV was first reported in 1971 by De Graciansky and Mery [4], although the term was first proposed in 2003 [5]. It manifests as numerous well‐demarcated, round or oval atrophying hyperhypopigmented macules or plaques with overlying fine‐scale lesions [2, 3]. To date, 34 cases have been reported in the literature in addition to the present case (Table 1). PV is typically diagnosed clinically with the help of noninvasive tools such as dermoscopy, Wood’s lamp examination, and direct microscopic examination using KOH preparation. However, due to the false‐negative results of Wood’s lamp examinations and direct microscopy, skin biopsy is valuable for excluding other possible diagnoses in equivocal cases [3].

**TABLE 1:** The characteristics and findings of the reported atrophying pityriasis versicolor.

No.	Author	Year of publication	Age/gender	Duration	Lesion/location on examination	Histopathological findings related to skin atrophy	History of topical steroid application (10/34)	Mx/duration	Outcome
1.	Tatnall FM [6]	1985	27/M	3 years	Telangiectatic erythematous/brown scaly macules with cutaneous atrophy over the chest and back	Not done	Yes	Clotrimazole 1% cream for 3 weeks	Atrophy resolved
2.			17/F	18 months	Brown scaly patches on her trunk were set into depressions	Not done	No	Selenium sulfide shampoo and clotrimazole 1% cream	Atrophy resolved
3.			39/M	20 years	Erythematous scaly atrophic lesions on his back, chest, arms and on the dorsa of his hands.	Not done	Yes	10% sodium thiosulfate solution	Atrophy resolved

4.	Crowson AN [7]	2003	17/F		Atrophic macules over back and shoulders	Dermal elastolysis	Yes	Ketoconazole	Atrophy resolved
5.			55/F		Atrophic macules over shoulders	Dermal elastolysis	No	Ketoconazole	Atrophy resolved
6.			19/F		Atrophic plaques over trunk and shoulders	Retiform effacement	No	Ketoconazole	Atrophy resolved
7.			57/M		Atrophic plaques over trunk and shoulders	Retiform effacement	No	Ketoconazole	Atrophy resolved
8.			21/M		Atrophic patches over anterior chest	Retiform effacement	No	Ketoconazole	Atrophy resolved
9.			72/F		Atrophic macules over forearm	Retiform effacement	No	Ketoconazole	Atrophy resolved
10.			58/F		Granulomata over eyelid, cheek, and nose	Retiform effacement	No	Ketoconazole	Atrophy resolved
11.			73/M		Atrophic macules over forearm	Retiform effacement	No	Ketoconazole	Atrophy resolved
12.			59/M		Atrophic macules (unspecified site)	Retiform effacement	Yes	Ketoconazole	Atrophy resolved
13.			22/M		Atrophic macules over left arm	Retiform effacement	No	Ketoconazole	Atrophy resolved
14.			25/F		Atrophic macules reticulated over upper back	Retiform effacement	No	Ketoconazole	Atrophy resolved
15.			72/F		1–3 cm atrophic salmon‐colored macules over back and shoulder	Retiform effacement	No	Ketoconazole	Atrophy resolved

16.	Romano C [5]	2005	49/F	7 years	Roundish, achromatic, some of them atrophic, asymptomatic patches	Dermal elastolysis	No	Topical imidazole and systemic itraconazole 100 mg daily for 15 days	Atrophy persists

17.	Yang YS [8]	2010	50/M	2 years	Multiple scaly erythematous atrophic macules and plaques	Focal thinning of the *epidermis* and dermal elastolysis	Yes	Oral itraconazole 200 mg/day for 2 weeks and flutrimazole ointment.	Atrophy improved

18.	Tellechea Ö [9]	2012	35/M	2 months	Asymptomatic, erythematous, slightly atrophic, well circumscribed small patches over the back	Flattening of rete ridges	No	Itraconazole (100 mg/day, 6 weeks)	Atrophy improved

19.	Ahn JJ [10]	2013	27/M	1 year	Confluent, relatively well‐defined, erythematous macules and patches on the neck, shoulders, back, and chest with atrophic changes	Focal thinning of the *epidermis* And dermal elastolysis	No	Oral itraconazole 200 mg and topical flutrimazole for 4 weeks	Atrophy resolved

20.	Cullingham K [11]	2014	47/M	—	Pink, atrophic, scaly plaques over the back	Not done	Yes	Systemic ketoconazole	Atrophy resolved
21.	Moon SY [12]	2016	35/M	3 years	Multiple depressed brownish macules and patches on the back	Dermal elastolysis	Yes	Topical amorolfine ointment	Atrophy improved
22.	Haiduk *J* [13]	2016	66/M	6 months	Red/violaceous or skin‐colored, partly coalescing, round to oval, atrophic, slightly depressed macules	Flattening of the rete ridges	Yes	Topical ketoconazole	Atrophy resolved

23.	Levy JM [14]	2017	56/M	2 years	Yellowish‐brown, slightly atrophic patches in the right axilla	Focal epidermal attenuation and dermal elastolysis	No	Single dose of ketoconazole 400 mg orally as well as topical ketoconazole cream.	Atrophy resolved
24.			50/M	Few years	Hyperpigmented, slightly atrophic plaques that were widespread over the body and fine scale over the trunk, arms, buttocks, and anterior thighs		No	Ketoconazole 400 mg orally once and then repeated after 2 weeks	—
25.			30/F	6 months	Pinkish‐brown atrophic patches on her neck, chest, abdomen, pubic area, and upper arms		No	Ciclopirox shampoo, glycolic acid (15%) lotion and later switched to ketoconazole (2% cream)	Atrophy resolved
26.			74/M	—	Atrophic, scaly plaques that were light‐brown over chest, neck, and arms		No	Ketoconazole 400 mg on Days 1 and 8, as well as ciclopirox shampoo daily	Atrophy resolved
27.			28/F	—	Annular, scaly, atrophic plaques that photo‐distributed on the back and extremities		No	—	—
28.			41/M	—	Hyperpigmented patches of unknown duration on the buttocks		No	—	—

29.	Marinello *E* [15]	2017	42/F	2 months	Faintly erythematous scaly roundish depressed patches all over the back from the shoulders to the lumbar area and on both sides of the neck	Not done	No	Topical ketoconazole 2% cream over a 6‐week	Atrophy resolved
30.	Allegue F [16]	2018	28/M	—	Circular hypopigmented and pink areas on the back and the extensor surface of the upper right limb with depressed and atrophic in appearance.	Flattening of the rete ridges diminished elastic fibers	Yes	Itraconazole 200 mg/d for 7 days and topical flutrimazole for 1 month.	Atrophy resolved
31.	Chang YM [17]	2021	45/F	Several months	Multiple erythematous, slightly scaly, depressed plaques, which were scattered or confluent on the back with lower back predominance	Flattening of the rete ridges and dermal elastolysis	No	Topical ketoconazole 2% cream twice daily	Atrophy improved
32.	Balakrishnan KD [18]	2022	39/F	3 months	Numerous hyperpigmented macules and patches with fine scaling and central atrophy over the anterior aspect of the left leg, lateral aspect of the thigh, medial aspect of the left arm, left breast and left side of the abdomen.	Atrophic *epidermis* and dermal elastolysis	yes	Oral itraconazole 100 mg twice daily along with topical ketoconazole 2% application twice daily. 2.5% selenium sulfide	Atrophy improved
33	Quazi S [19]	2024	29/M	3 months	Well‐defined multiple hypopigmented macules of varying sizes with fine scales were observed on the patient’s chest, shoulders, and arms	Flattening of rete ridges	No	2% topical ketoconazole and 2.5% selenium sulfide along with 100 mg oral itraconazole twice daily	Atrophy resolved
34.	Our case	2024	15/F	2 years	Multiple well‐demarcated brown scaly atrophic macules and patches over the upper back, anterior shoulder and upper chest	Not done	No	Ketoconazole 2% shampoo 3 times/week. terbinafine 1% cream twice/daily and itraconazole 200 mg PO once daily for 1 month.	Atrophy resolved

In most reported cases (Table 1), the histopathology has revealed classical findings of PV, such as hyperkeratosis and “spaghetti and meatballs” appearance of the periodic acid–Schiff (PAS) stain. In addition, it has shown flattening of the rete ridges and/or elastolysis in Verhoeff–van Gieson (VVG) or Orcein stains, which are compatible with the clinically observed skin atrophy. Of the 15 patients who showed alteration of the elastic fibers, 5 patients used topical steroids before a biopsy was taken.

The treatment of atypical PV variants can be challenging [4]. Moreover, in tropical regions characterized by warm temperatures and high humidity year‐round, PV becomes resistant to topical treatment with high recurrence in some cases. For this reason, we treated our patient with oral itraconazole for 1 month and used topical therapy for a longer duration. As shown in Table 1, patients whose skin lesions had not resolved completely were treated with systemic antifungal agents alone or in combination with topical antifungal medications for 2 weeks.

Two hypotheses have been proposed in the literature to explain the pathophysiology of skin atrophy. The first one involves two possible mechanisms. The first is a direct mechanism involving the innate immune response to *Malassezia* antigens, which increases the production of proinflammatory cytokines such as IL‐1 and TNF‐β. This process inhibits the nuclear factor (NF)‐κB pathway and results in keratinocyte apoptosis and decreased proliferation, which would explain the retiform effacement that has been observed in 24 cases (Table 1). The other mechanism occurs indirectly through a delayed hypersensitivity reaction, in which Type‐1 T‐helper cells recruit histocytes and lead to an increase in elastase production. This mechanism would explain the fragmentation and decrease of elastic fibers in 15 of the reported patients (Table 1) [5, 7].

The second hypothesis is linked to longer use of topical steroids, which could lead to epidermal atrophy and inhibit dermal collagen synthesis [8]. However, out of 34 reported cases, only 10 patients were using topical steroids. In fact, for 7 out of 10 patients who underwent skin biopsy, the results showed no alteration in collagen fibers (Table 1), which is not consistent with this latter hypothesis.

Atrophic skin lesions are one of the rare presentations of PV. In this case report, we have highlighted the importance of considering the diagnosis of atrophying PV along with other cutaneous diseases that manifest with such skin lesions, including anetoderma, atrophoderma of Pasini and Pierini, sarcoidosis, parapsoriasis, and mycosis fungoides. Quick and noninvasive examination tools, such as dermatoscopy, Wood’s lamp, and direct microscopic examinations using KOH preparation, are useful in establishing a diagnosis. However, the latter two tools may yield false‐negative results, so a punch biopsy may be needed in inconclusive cases.

Dermatoscopy typically shows a structureless hypopigmented or hyperpigmented patch with fine scales, which are more prominent in perifollicular and skin‐furrow areas. Reticular lines are another finding in darker skin types. Peripheral hypopigmentation that surrounds hyperpigmented patches or is located within them is referred to as the contrast halo sign [4, 19].

This study has certain limitations. The atrophy was diagnosed based on clinical judgment alone, and histopathology with special stains was not performed, which represents a limitation. We do not recommend invasive testing to confirm the diagnosis of PV; rather, such testing may be considered only for the objective diagnosis of atrophy. Furthermore, we do not recommend prolonged itraconazole therapy in the routine management of PV cases.

This case report and literature review were meant to shed light on the atrophying variant of PV, of which 34 cases have been reported, including the present case. We suggest that this variant be listed as part of the differential diagnosis for atrophying skin lesions, especially if skin lesions have a seborrheic distribution. Furthermore, we encourage clinicians to consider Wood’s lamp, KOH microscopy, and dermatoscopy examinations before proceeding to skin biopsy.

## Funding

The authors have received no funding to conduct this study.

## Consent

Patient consent was obtained for publication of this report.

## Conflicts of Interest

The authors declare no conflicts of interest.

## Data Availability

All relevant data supporting the findings of this case report are included within the article. No additional data are available.
